# Advancing Microplastic and Nanoplastic Toxicity Assessment: Insights from Human Organoid Models

**DOI:** 10.3390/bioengineering13030309

**Published:** 2026-03-06

**Authors:** Lingling Ge, Yingying Lan, Jing Gong, Xue Gao, Francesco Faiola, Shaocheng Zhang, Minghui Li

**Affiliations:** 1 Department of Laboratory Medicine, The Second Affiliated Hospital of Chengdu Medical College (Nuclear Industry 416 Hospital), Chengdu 610051, China; 2 Southwest Hospital, Third Military Medical University (Army Medical University), Chongqing 400038, China; 3 Department of Infectious Diseases, Institute for Viral Hepatitis, The Second Affiliated Hospital, Chongqing Medical University, Chongqing 400016, China; 4 Key Laboratory of Biorheological Science and Technology, Ministry of Education, College of Bioengineering, Chongqing University, Chongqing 400045, China; 5 State Key Laboratory of Environmental Chemistry and Ecotoxicology, Research Center for Eco-Environmental Sciences, Chinese Academy of Sciences, Beijing 100085, China; 6 Institute of Chemistry and Biochemistry, Freie Universität Berlin, 14195 Berlin, Germany

**Keywords:** human organoids, microplastics, nanoplastics, toxicity assessment, organoid toxicology

## Abstract

Evidence has demonstrated that microplastics (MPs) and nanoplastics (NPs) exerted toxic effects on animal models; our understanding of their potential toxicity to human health remains limited due to the lack of human-relevant in vitro models. Human stem cell-derived organoids are sophisticated and multicellular structures that could effectively mimic the key features of native organs and tissues. To date, human organoids have been widely used in toxicology. This study reviews the current application of human organoids in the toxicity assessment of MPs and NPs. Current limitations and future perspectives are discussed. Cutting-edge technologies combined with organoids are expected to provide new insights for illustrating the potential toxicity of MPs and NPs.

## 1. Introduction

Microplastics (MPs, <5 mm in size) and nanoplastics (NPs, <1 μm in size) are minute plastic particles that have garnered significant attention due to their widespread presence and potential harm to ecosystems and human health [1,2,3,4,5]. A growing body of evidence indicates that human exposure to micro- and nanoplastics (MNPs) is unavoidable, originating from various sources, including food, water, ambient air, and indoor dust [6,7]. Although evidence has proven that exposure to MNPs poses threats to animal health, our understanding of their potential risks to human health remains limited. The toxicity assessment of MNPs is predominantly hindered by the scarcity of epidemiological data on human exposure and the absence of suitable experimental models that accurately replicate human responses to MNPs. Organoids are small three-dimensional cell structures grown from stem cells (embryonic stem cells or induced pluripotent stem cells) and can mimic the real organs in terms of cellular complexity, architecture, and function. Organoid models show great promise in advancing biomedical research and regenerative medicine. Recently, growing evidence has advocated the application of three-dimensional human organoids in MNP toxicity assessment.

## 2. Human Exposure to MNPs

MNPs primarily result from the breakdown of plastic products such as containers, bags, and clothes, even disposable face masks [2,8]. Once plastic waste enters the environment, it undergoes physical, chemical, and biological degradation, thus releasing plastic debris (e.g., MPs and NPs) into aquatic, terrestrial, and atmospheric ecosystems [9]. Consequently, MP-/NP-contaminated foods, water, and ambient air pose significant threats to human health [10]. Human exposure to MNPs occurs not only through oral ingestion and inhalation but also via skin contact, deliberately introduced into cosmetics and abrasive cleaners [11,12,13]. Currently, the presence of MNPs has been detected in human samples, such as colonic and lung tissues, feces, blood, brain, and even retina, suggesting a potential link between their levels or occurrence and the risk of human diseases [14,15,16,17,18,19,20]. For instance, in patients with inflammatory bowel disease (IBD), the concentration of fecal MPs showed a positive correlation with the severity of IBD, implying a potential association between MP exposure and the progression of intestinal diseases [21]. However, the underlying mechanisms of MP-induced injury are still unknown. Furthermore, the number of MPs in thrombi was positively correlated with blood platelet levels, raising concerns about an increased risk of cardiovascular diseases associated with MP exposure [22,23]. Although larger particles cannot breach physiological barriers, the intake of MPs has been shown to disrupt the integrity of epithelial and endothelial layers, enabling their translocation into the circulatory system and subsequently posing threats to various organs, such as the liver, kidney, and brain [24,25,26,27,28,29]. Moreover, plastic particles originating from maternal exposure can cross the placental barrier, posing risks to embryonic development [30,31]. While these findings currently represent hypotheses regarding the potential involvement of MNP exposure in human health, the direct damage of MNPs to human systems/organs needs to be further illustrated.

**Figure 1:**
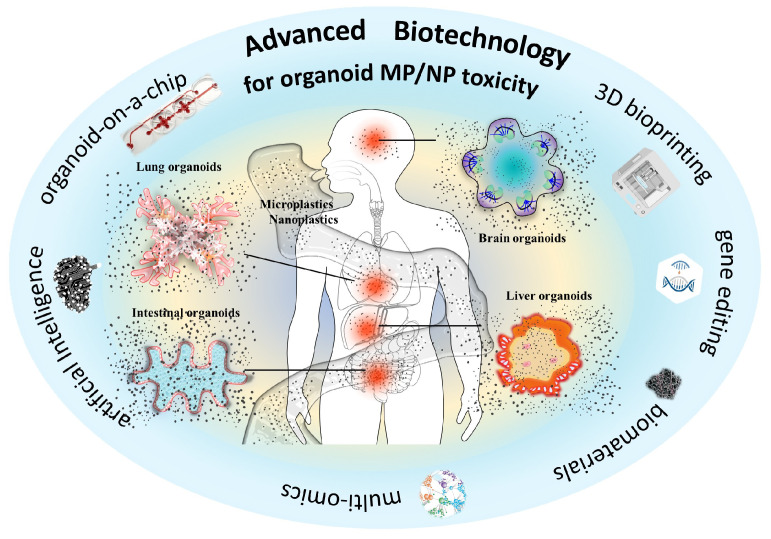
Advanced biotechnology aims to promote the assessment of the toxicity of micro- and nanoplastics based on human organoids.

Early toxicity assessments of MPs were often conducted using marine and aquatic animal models, including fish, shrimp, mussels, and oysters [32,33,34]. Exposure to MNPs in animals led to alterations in intestinal flora, metabolic disorders, oxidative stress, and immune dysfunction [35,36,37]. Similarly, MP exposure demonstrated toxicity to reptiles and mammals [38,39,40]. In addition to animal models, MNP exposure caused toxic effects on human cell lines by inducing oxidative stress and inflammation, adversely impacting cellular health and viability [41,42]. Although great efforts have been made to assess the toxicity of MNPs and investigate the underlying molecular mechanisms, the reliability of the conclusions is controversial to a certain extent due to interspecies variations and the absence of a physiologically relevant microenvironment for humans. The emergence of “stem cell toxicology” presents a promising avenue for assessing toxicity related to human health without relying on animal models [43]. This groundbreaking approach paves the way for using stem cell-derived organoids in toxicology. Human organoids, derived from human stem cells, possess intricate multicellular structures and can mimic the functionality of native organs in vivo. Organoids have been established for various organ systems, including, but not limited to, the gastrointestinal tract, stomach, kidney, liver, lung, thyroid, and brain [44,45]. Human organoids serve as invaluable models for next-generation toxicology tests, offering alternative methods to reduce our dependence on animal experimentation. Consequently, organoid-based toxicology holds great promise for gaining a more comprehensive understanding of the toxicity of MNPs on human health (Figure 1).

## 3. Human Organoids for Toxicity Assessment of MNPs

### 3.1. Human Organoids Applied for Assessing MP Toxicity

**Figure 2:**
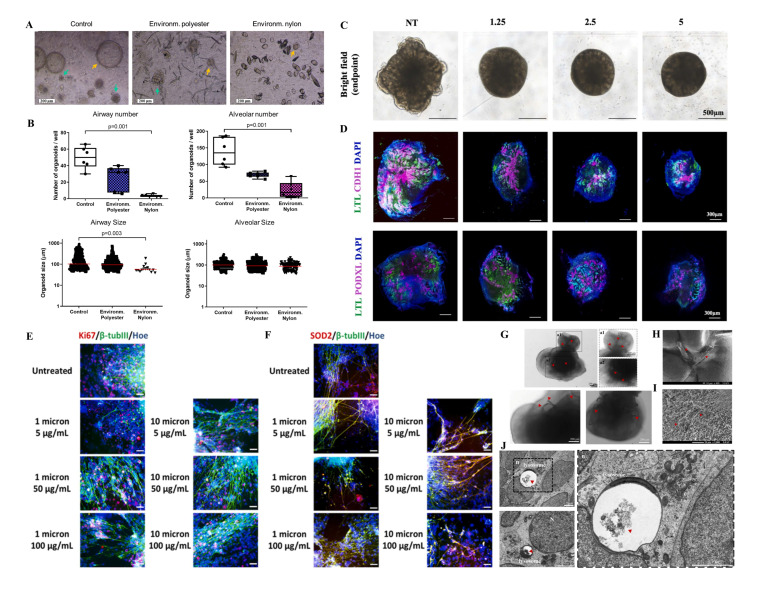
Human organoids applied for microplastic toxicity assessment. (**A**,**B**): Effects of MP fibers on lung organoids. Reproduced with permission [47]. (**C**,**D**): MPs disrupted kidney organoid development. Reproduced with permission [53]. (**E**,**F**): Exposure to MPs disrupted cortical organoid growth. Reproduced with permission [54]. (**G**–**J**): Face mask-derived MPs were internalized by retinal organoids. Reproduced with permission [55].

As ambient MPs can enter the human body mainly through ingestion, inhalation and dermal contact, researchers have generated respiratory, gastrointestinal, and skin organoids to uncover their potential toxicity (Table 1) [46,47,48,49,50]. For instance, human airway organoids (hAOs) were established to evaluate the toxic effects of MP fibers (MPFs) derived from synthetic clothes and fabrics, at concentrations of 1, 10, and 50 µg/mL [46]. These MPFs were observed with the organoids and were found to impact the expression of *SCGB1A1*, a gene closely associated with club cell functionality. However, MPFs did not inhibit organoid growth significantly or induce substantial inflammation or oxidative stress. The presence of MPFs within the organoid system raises concerns about their potential long-term impact on the repair process of lung epithelial cells [46]. Similarly, human alveolar and airway-type organoids were used to investigate the toxic effects of MPFs leaching from nylon 6,6 and polyethylene terephthalate (polyester) (Figure 2A,B) [47]. Nylon microfibers, more than polyester, inhibited the development of AOs. Although these findings demonstrate that MPs cause adverse effects on cellular events in hAOs, the potential impacts of MPs on lung function are still unknown. Moreover, the possible translocation of MNPs across the airway epithelium needs to be investigated in further studies. Given the critical roles of immune cells in respiratory microenvironments, immunized respiratory organoids are expected to reveal human responses to MNP insults.

In parallel, gastrointestinal organoid models, such as human colon and intestinal organoids, were developed to elucidate the toxicity of MPs to the human gastrointestinal system [50,56,57]. Human colon organoids exposed to small MP particles exhibited reduced viability and increased expression of genes related to inflammation, apoptosis, and immunity [50]. Chen et al. used human intestinal organoids to develop epithelia to mimic the cell complexity and functions of native tissue [58]. After exposure to MPs, the Microfold cells acted as sensors, capturers and transporters of larger-sized particles. The internalization of particles by epithelial cells exhibited size-, concentration-, and time-dependent manners. High concentrations of particles were found to trigger the secretion of a panel of inflammatory cytokines linked to human IBD. These findings underscore the potential of gastrointestinal organoids in predicting and assessing the gastrointestinal toxicity of MNPs in humans. It is worth noting that the microenvironment of the gastrointestinal system cannot be neglected due to the internal conditions in terms of temperature, pH, microbiota, and digestive juice (e.g., bile acids) that could influence the stability, uptake, and transport of MNPs. Given that ingested MNPs could cause damage to the gastrointestinal epithelium and then cross the epithelial barrier, gastrointestinal organoids combined with a vascular system, or vascularized gastrointestinal organoids are strongly recommended to mimic human exposure. Different gastrointestinal organs could be exposed to diverse shapes and concentrations of MNPs, so experiments need to be conducted based on human exposure conditions.

Once ingested or inhaled MNPs penetrate biological barriers, and they may disseminate into the circulatory system, posing threats to various organs. Human-originated cardiac organoids have been generated to reveal the potential toxicity of MPs to the human heart. Human exposure levels of polystyrene-MPs (PS-MPs) caused oxidative stress, inflammatory response, apoptosis, and collagen accumulation, which were consistent with in vivo observation [51]. MPs caused pathological changes similar to myocardial hypertrophy. The aberrant changes in the expressions of hypertrophic-related genes (*MYH7B*, *ANP*, *BNP*, *COL1A1*) was accordant to the thickened cardiac structure and compressed atrial and ventricular cavities. Moreover, human liver, kidney, brain, and retinal organoids have been used to investigate the hepatotoxicity, renal toxicity, and neurotoxicity of MPs [52,53,54,55,59]. For instance, PS-MPs cause reactive oxygen species (ROS) generation, oxidative stress, inflammation response, and alteration in lipid metabolism, which may implicate liver steatosis and fibrosis [52]. MP exposure increased ROS and mitochondrial damage in nephron progenitors and reduced glycolysis by decreasing glucose transporters (GLUTs) and phosphofructokinase (PFK) activity, contributing to nephrogenesis disruption in human kidney organoids (Figure 2C,D) [53]. Exposure to MPs was found to cause a reduction in neural precursor cells and neuronal cells, damage cortical layer differentiation, and disrupt neuronal maturity with brain organoids (Figure 2E,F) [54]. Our previous study has proved that exposure to face mask-derived MPs disrupted anatomical structure development, differentiation, and neurogenesis in the retinal organoids (Figure 2G–J) [55]. Apart from inhalation and ingestion, dermal contact is also a potential exposure route. Although the skin acts as a highly effective barrier against MNP uptake, smaller MNPs can reach the deeper layer when the tight junction function is disrupted [60]. Hu et al. applied skin organoids to assess MP-induced skin toxicity [48]. The study proved that smaller MP sizes and ultraviolet A irradiation exhibited higher uptake and internalizations, potentially contributing to skin toxicity. Taken together, these studies collectively highlight the importance of assessing toxicity in human organoid models to gain critical insights into the impact of environmental MPs on human health. However, organoid-based MP toxicity assessment is still in its nascent stages.

### 3.2. Human Organoids Applied for Assessing NP Toxicity

**Figure 3:**
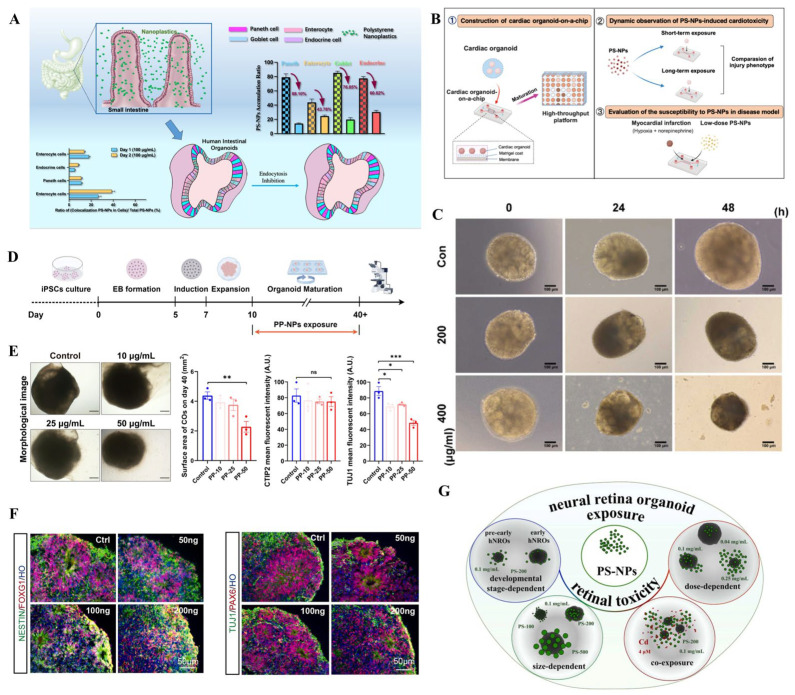
Human organoids applied for nanoplastic toxicity assessment. Human organoids applied for microplastic toxicity assessment. (**A**): Distinct accumulation of NPs in human intestinal organoids. Reproduced with permission [49]. (**B**): NP-induced cardiotoxicity based on cardiac organoid-on-a-chip. Reproduced with permission [64]. (**C**): NPs induced renal injury based on kidney organoids. Reproduced with permission [72]. (**D**,**E**): Exposure to NPs disrupted cerebral organoid development. Reproduced with permission [68]. (**F**): NPs impaired neural development in human cerebral organoids. Reproduced with permission [69]. (**G**): Retinal organoids were applied to illustrate the retinal toxicity of NPs. Reproduced with permission [71]. * *p* < 0.05; ** *p* < 0.01; *** *p* < 0.001; ns, not significant.

Evidence has shown that NPs are potentially more dangerous than MPs due to their smaller size [61,62]. NPs can penetrate biological barriers, reach and accumulate in vital organs, causing direct damage. To date, human organoid models have also been used to assess the potential toxicity of NPs (Table 2). For example, human intestinal organoid exposure to NPs of approximately 50 nm showed cytotoxic effects, including cellular apoptosis and inflammatory response, consistent with the toxicological responses observed in colon organoids [49]. Notably, the accumulation of NPs in goblet, Paneth, and endocrine cells demonstrated both time- and dose-dependent patterns (Figure 3A). To visualize the internalization of NPs in intestinal organoids, Okkelman et al. reported a novel approach combining intestinal organoid culture with live fluorescence lifetime imaging microscopy [63]. This method improved the sensitivity of fluorescence lifetime imaging microscopy over conventional intensity-based microscopy, enabling the tracing of fluorescent NPs in live intestinal organoids. Xuan et al. proved that three NPs (PS, PTFE, and PMMA) exposure reduced mitochondrial membrane potential, intracellular ROS accumulation and oxidative stress, and inhibited the AKT/mTOR signaling pathway of intestinal organoids [57]. Moreover, non-targeted metabolomics results confirmed that three types of NPs regulated fatty acid metabolism, nucleotide metabolism, necroptosis and autophagy pathways.

Cardiac organoids combined with chip technology were applied for cardiotoxicity evaluation of NPs through multiple dimensions, such as multi-stage observation (Figure 3B) [64]. Exposure to PS-NPs induced oxidative stress, inflammation, disruption of calcium ion homeostasis, and mitochondrial dysfunction in the early stage. Cardiac fibrosis emerged as a prominent feature after long-term exposure. Low-dose PS-NP exposure was found to aggravate the symptoms of hypoxia/norepinephrine-induced myocardial infarction. Moreover, NP-exposed cardiac organoids displayed aberrant contraction amplitude and frequency. Li et al. reported that PS-NP exposure disrupted the efficiency of cardiomyocyte differentiation from human embryonic stem cells, resulting in immature cardiomyocytes and smaller cardiac organoids with impaired contractility [65]. The reduced pluripotency of human embryonic stem cells could be caused by enhanced mitochondrial oxidative stress, activated P38/Erk MAPK signaling pathway, and blocked autophagy flux. These studies proved that NP exposure could pose threats to human heart development.

**Table 2:** Organoids applied to assess nanoplastic toxicity.

Organoids	NP Type & Size	Exposure	Toxic Effects	Toxic Mechanism	References
Intestinal organoid	polystyrene (50 nm)	2 days (10 and 100 µg/mL)	induced cellular apoptosis and inflammatory response	elevated NF- NF-κB, p65, IL-8, and ROS	[49]
Intestinal organoid	polystyrene, polytetrafluoroethylene, and polymethyl methacrylate (100 nm)	3 days (50 µg/mL)	reduced mitochondrial membrane potential, intracellular ROS accumulation and oxidative stress	inhibited the AKT/mTOR signaling pathway	[57]
Lung organoid	polystyrene (40 nm)	12, 24, 48, and 72 h (100 µg/mL)	restricted organoid growth and caused oxidative damage	increased expression of *Cldn4* and decreased expression of *Sftpc* and *Pdpn*	[66]
Lung organoid	polystyrene (250 nm)	14 days (1 and 10 µg/cm^2^)	the internalization of PS-NPs	increased phosphorylation of both AKT and ERK	[67]
Cardiac organoid	polystyrene (40 nm)	10 days (30–150 μg/mL)	induced oxidative stress, inflammation, disruption of calcium ion homeostasis, and mitochondrial dysfunction	activated pathways associated with collagen and extracellular matrix dynamics	[64]
Cardiac organoid	polystyrene (20 nm)	24 h (5 or 20 μg/mL)	disrupted the efficiency of cardiomyocyte differentiation; impaired contractility	involved in autophagy and ROS/p38/Erk MAPK signaling pathways	[65]
Cerebral organoid	polypropylene (100 nm)	day 10–40 (10, 25, and 50 µg/mL)	reduced growth and neuronal differentiation; downregulated neuronal markers	disrupted neuroactive ligand- receptor interaction pathway	[68]
Cerebral organoids	polystyrene (<50 nm)	day 16 to day 24 (50, 100, 200 ng/mL)	increased cell death; decreased cell differentiation and neuronal activity	induced mitochondrial impairment; disrupted neuronal calcium activity	[69]
Brain organoid	polystyrene (50 nm)	7 days (50 and 100 µg/mL)	inhibited neuronal synaptogenesis	induced endoplasmic reticulum stress; disrupted cholesterol homeostasis	[70]
Retinal organoid	polystyrene (100, 200, and 500 nm)	14 days (0.04, 0.1, and 0.25 mg/mL)	decreased organoid size, reduced cell proliferation, increased apoptosis, and altered gene expression profiles	disrupted axon guidance, anatomical structure development, differentiation, and neurogenesis	[71]
Kidney organoid	polystyrene (100 nm)	48 h (200, 400, and 800 μg/mL)	Inhibited organoid growth; significant cell detachment	disrupted proliferation and differentiation	[72]

Recently, Yang et al. used lung organoids to investigate the potential lung injury induced by PS-NPs [66]. Exposure to PS-NPs over 24 h reduced lung organoid-forming efficiency with decreased size and transparency. PS-NP exposure could induce oxidative damage by overproducing ROS. Similar to in vivo, exposure to NPs causes aberrant transitional cell behavior. Ernhofer et al. applied patient-derived lung organoids to investigate the internalization of PS-NPs [67]. This study confirmed that PS-NPs can penetrate multiple cellular barriers. Increased phosphorylation of both AKT and ERK was observed in NP-exposed organoid, which was consistent with their expression in non-malignant BEAS-2B cells. This data suggests that PS-NP-induced cellular response could potentially contribute to malignant transformation. Chen et al. investigated the toxicity of PS-NPs to kidney organoids (Figure 3C) [72]. Similar to MPs, NPs were found to disrupt the growth and development of kidney organoids. PS-NPs were internalized into the inner part of kidney organoids and triggered lamellar apoptosis and detachment of organoid cells. Compared with immortalized cell lines, kidney organoids exhibited a heightened sensitivity to NPs.

Exposure to polypropylene-NPs (PP-NPs) was found to reduce the growth of cerebral organoids (Figure 3D,E) [68]. Significantly decreased expression of neuronal markers such as *TUJ1*, *MAP2*, *RBFOX3*, and *PAX6* was found in cerebral organoids exposed to 50 µg/mL PP-NPs, suggesting that PP-NPs impaired neuronal differentiation. NP exposure induced differentially expressed genes that were closely associated with the neuroactive ligand-receptor interaction pathway. Tao et al. found that PS-NPs can intrude into the mitochondria of cerebral organoids, causing mitochondrial structural and functional alteration, resulting in increased cellular death and neural differentiation (Figure 3F) [69]. Moreover, neuronal calcium and electronic activity (amplitude of action potential) were downregulated in NP-exposed cerebral organoids. Tian and colleagues proved that PS-NPs induced astrocytic endoplasmic reticulum stress, impaired cholesterol de novo synthesis and apolipoprotein-mediated transport, ultimately inhibiting neuronal synaptogenesis in whole-brain organoids [70]. Given that NPs can penetrate the blood-retina barrier, accumulate in the retina tissue, and impair retina, resulting in retinal dysfunction in mice, Gao et al. investigated the neurotoxic effects of PS-NPs on retinal organoid development [71]. Exposure to PS-NPs decreased organoid size, reduced cell proliferation, increased apoptosis, and disrupted gene expression profiles of retinal organoids (Figure 3G). Smaller PS-NPs were found to cause more severe neurotoxicity than larger PS-NPs. All sizes of NPs might exert similar neurotoxic effects by disrupting axon guidance, anatomical structure development, differentiation, and neurogenesis. This study proved that the retinal toxicity of PS-NPs showed size-, dose-, and development-dependent manners. These data indicate that exposure to NPs disrupted neural organoid development.

## 4. Current Limitations and Future Perspectives

Although human organoids hold great promise in toxicity assessment of MNPs, several issues need to be considered in future studies. First, MNPs induce toxicity in a dose-dependent manner, while the exact exposure levels in human organs remain unknown. Given the presence of human biological barriers, the concentration of MNPs that end up in the target organs is minimal, especially in the brain [17]. Therefore, large-scale epidemiological studies are urgently demanded for organoid-based study designs. Second, the realistic microenvironments in human internal organs play critical roles in the interaction between MNPs and tissues. The dynamic interplay between MNPs and immune microenvironments (e.g., macrophage, microglia), physical microenvironments (e.g., gastrointestinal motility), microbial microenvironments (e.g., gut and lung microbiota), body fluids (e.g., digestive juice, mucus), and enzymes could influence the fate of MNPs in the human body. Therefore, engineering the intricate microenvironments into human organoids is essential for organoid-based toxicology. Third, humans are continuously exposed to MNPs from contaminated foods, water, and air during their lifetime, leading to chronic toxic effects. Studies on long-term and low-dose exposure to MNPs are necessary for elucidating the potential toxicity of MNPs. Future studies need to illustrate the size- and dose-dependent effects of MNPs on human organoids. Moreover, challenges in long-term organoid culture need to be addressed. Although MNP exposure caused alterations in cellular events, morphologies, or even functions of human organoids, how or whether these injuries are associated with human diseases is largely unknown. Commercial MNPs have been widely used in most toxic studies. However, these MNPs cannot mimic environmental and human-relevant exposure due to their simple shapes and components. For instance, humans would be exposed to teabag-derived MNPs, which are nylon and polyethylene terephthalate [73]. Therefore, the toxicity of consumer plastic product-derived MNPs should be revealed based on human organoids. Given that NPs could pose more serious biological hazards than MPs due to their smaller size, more studies on organoid-based NP toxicity assessments are necessary [71].

In addition to the MNP itself, the interaction between MNPs and biological systems is profoundly influenced by the formation of a protein corona [74]. Protein corona is a layer of adsorbed biomolecules that confers a new biological identity to the particles [75]. Evidence from traditional cell cultures and animal models has shown that the formation of protein corona alters cellular uptake, intracellular translocation, and toxicity [76,77,78,79]. However, limited data reported its roles in MNP-induced toxicity in organoids. A recent study proved that MP-induced toxicity correlated with protein corona composition based on an induced pluripotent stem cell-derived intestinal epithelial cell model [80]. Advanced human organoid models offer a promising platform to investigate these corona-mediated effects in a physiologically relevant context [81]. Organoids can recapitulate multi-cellular architecture and functions, enabling assess how the protein corona influences MNP translocation, accumulation, and chronic toxicity across different organ systems. Future studies combining organoid technology with corona characterization will be crucial for deciphering the mechanistic role of the protein corona in MNP-induced health effects and for improving environmental health risk assessment.

To fully harness the potential of human organoids in assessing MNP toxicity, cutting-edge technologies have been employed to tackle challenges related to the variability, fidelity, and maturation of organoids [82,83,84] (Figure 1). For example, organoid vascularization and immunization strategies aim to enhance their physiological relevance. The presence of vasculature and immune cells can enable the transport of MNPs within organoids. Given that ingested and inhaled MNPs can pass through biological barriers to reach the circulatory system and translocate to multiple organs, multi-organoids on a chip device allows their inter-organ crosstalk under circulatory perfusion conditions, providing a great opportunity for studying the translocation of MNPs between different organoids. More human organoid models, such as testicular and ovarian organoids, could be applied to fully reveal the toxicity of MNPs. The chip device combined with multi-organoids might help to decipher the systemic toxicity of MNPs to humans [85,86]. Moreover, organoid-on-a-chip platforms equipped with advanced imaging and analytical techniques are expected to dynamically monitor the uptake, accumulation, and distribution of MNPs. Artificial intelligence (AI) also provides available algorithms that could automate the histopathological evaluation of toxicity in organoids [87,88]. Integration of organoid technology and AI is expected to provide new insight into understanding the toxicity of MNPs. Recently, 3D bioprinting of organoids has been used for toxicity studies, particularly in assessing the toxicity of nanoparticles [89,90]. Thus, 3D bioprinting-based organoid models are expected to promote the toxicity assessment of MNPs.

## 5. Conclusions

In summary, the applications of human organoids are undeniably setting the stage for assessing the toxicity of MPs and NPs to humans. However, the application of human organoids to assess MP/NP toxicity is a nascent field. Cutting-edge technologies like AI and 3D bioprinting are being used to advance human organoid development. Consequently, these improved organoid models are expected to significantly enhance our understanding of the potential effects of MPs/NPs. To better inform the design of organoid exposure experiments, there is a need for more epidemiological studies that characterize human exposure scenarios (realistic exposure) and identify early toxicity endpoints. By integrating advanced analytical methodologies with human-relevant exposure and diverse MP/NP properties (e.g., type, shape, size, surface charge) can comprehensively reveal their potential toxicity. As MPs/NPs may pose threats to organ development, therapeutic interventions targeting specific toxic mechanisms should be developed to mitigate the risks of exposure.

## Figures and Tables

**Table 1:** Organoids applied to assess microplastic toxicity.

Organoids	NP Type & Size	Exposure	Toxic Effects	Toxic Mechanism	References
Airway organoid	polyester fibers from the air filter of a dryer machine	7 days (1, 10, and 50 µg/mL)	did not inhibit organoid growth	reduction of *SCGB1A1*	[46]
Lung organoid	Nylon MPs (1–5 μm and 5–10 μm)	7 days (16–39 μg/mL)	impaired organoid growth	upregulation of *Hoxa5*	[47]
Cardiac organoid	polystyrene (1 μm)	day 18-day 21 (0.025, 0.25 and 2.5 µg/mL)	increased oxidative stress, inflammatory response, apoptosis, and collagen accumulation	aberrant expression of hypertrophic-related and cardiac-specific genes	[51]
Liver organoid	polystyrene (1 μm)	48 h (0.25, 2.5 and 25 μg/mL)	Inducing ROS generation, oxidative stress, inflammation response, and alteration in lipid metabolism	increase the expression of hepatic HNF4A and CYP2E1	[52]
Kidney organoid	polystyrene (1 μm)	2 days (1.25, 2.5, and 5 μg/mL)	reduced size and abnormal tubular structures; increased level of mitochondrial ROS and DNA damage	glycolysis inhibition	[53]
Cortical spheroid	polystyrene (1 µm and 10 µm)	day 4–30 (100, 50, and 5 µg/mL)	short-term exposure promoted proliferation; log-term exposure reduced cell viability; DNA damage	elevated *SOD2* gene expression; downregulated *TUBB3* and *TBR1*/*TBR2* expression	[54]
Retinal organoid	MPs derived from face masks (0.2 mm filtering)	21 days (0.01, 0.1, 0.5, and 1 mg/mL)	disrupted the growth and development of retinal organoids	disordered neurogenesis, anatomical structure morphogenesis, and axon guidance	[55]
Skin organoid	polystyrene (100 and 500 nm)	12 h co-culture (100 μg/mL)	size-dependent MP uptake and ROS induction	size-dependent expression of oxidative stress-associated genes (*p53* and *Bax*)	[48]

## Data Availability

No new data were created or analyzed in this study.
